# The association between arterial stiffness and socioeconomic status: a cross-sectional study using estimated pulse wave velocity

**DOI:** 10.1186/s40885-024-00284-7

**Published:** 2024-10-01

**Authors:** Hack-Lyoung Kim, Soonil Kwon, Hyun Sung Joh, Woo-Hyun Lim, Jae-Bin Seo, Sang-Hyun Kim, Joo-Hee Zo, Myung-A Kim

**Affiliations:** Division of Cardiology, Department of Internal Medicine, Seoul Metropolitan Government-Seoul National University Boramae Medical Center, Seoul National University College of Medicine, 5 Boramae-ro, Dongjak-gu, Seoul, 07061 Korea

**Keywords:** Educational status, Income, Pulse wave analysis, Socioeconomic status, Vascular stiffness

## Abstract

**Background:**

The impact of socioeconomic status (SES) on arterial stiffness remains unclear. This study aimed to explore the association between both personal and household income, as well as education level, and estimated pulse wave velocity (ePWV).

**Methods:**

A total of 13,539 participants (mean age 52.9 ± 16.7 years; 57.1% women) from the Korean National Health and Nutrition Survey database were analyzed. For SES variables, information on personal and household income and education level was collected using standardized questionnaires.

**Results:**

The ePWV did not show significant differences across groups categorized by individual income levels (*P* = 0.183). However, there was a noticeable trend of decreasing ePWV with increasing household income levels (*P* < 0.001). Additionally, ePWV demonstrated a significant negative correlation with higher education levels, indicating that ePWV decreased in groups with higher educational attainment (*P* < 0.001). In multiple linear regression analyses, both household income (*β* = -0.055; *P* < 0.001) and education level (*β* = -0.076; *P* < 0.001) were negatively associated with ePWV, even after adjusting for potential confounders.

**Conclusions:**

Lower household income and lower education levels were associated with higher ePWV, providing further evidence of the influence of SES on arterial stiffness.

**Graphical abstract:**

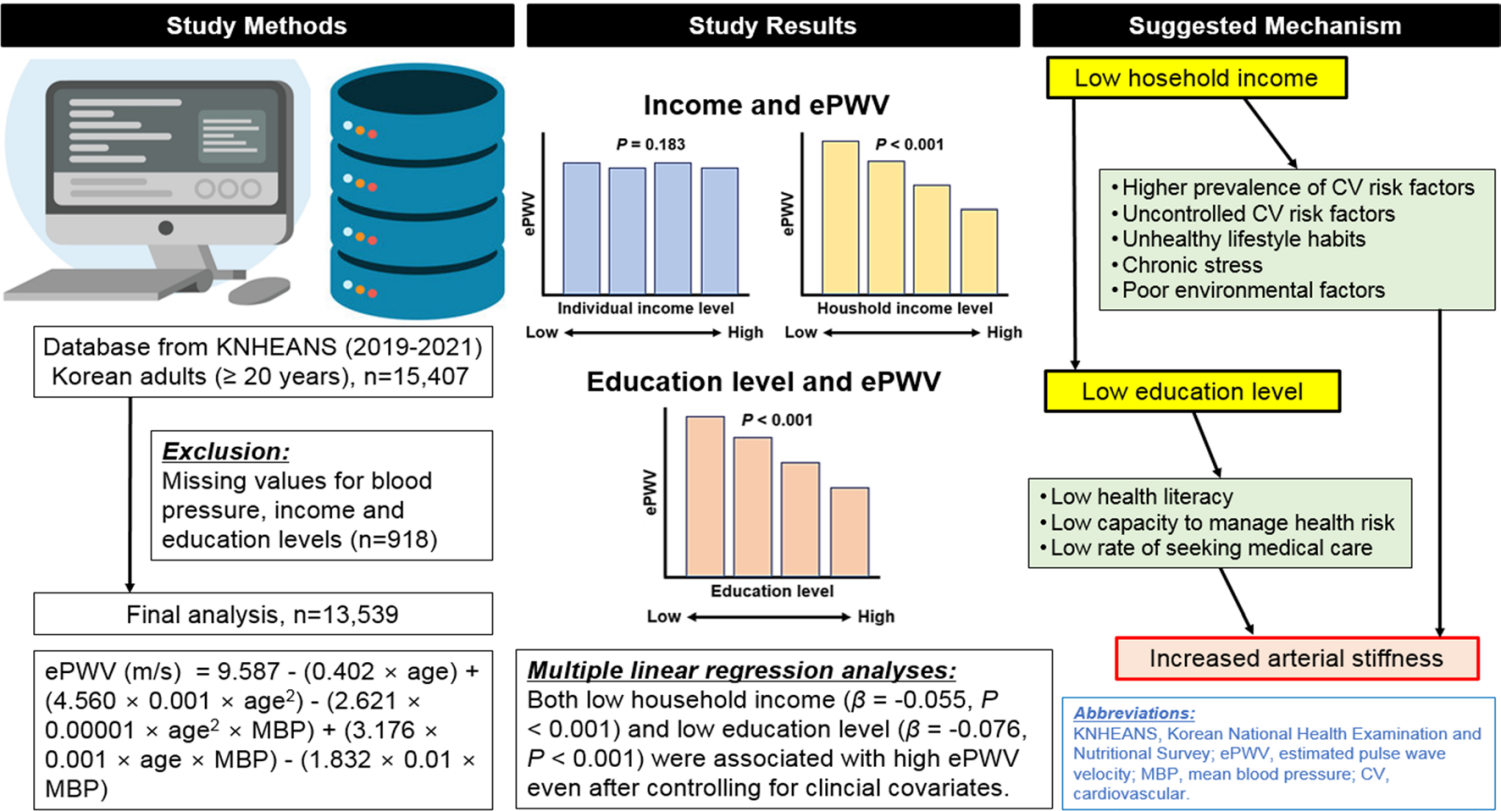

**Supplementary Information:**

The online version contains supplementary material available at 10.1186/s40885-024-00284-7.

## Introduction

Socioeconomic status (SES) significantly influences cardiovascular disease (CVD) risk, with individuals of lower SES experiencing higher incidence of CVD and mortality [1]. Various mechanisms link SES to CVD, including differential access to healthcare, unhealthy lifestyles due to limited resources, chronic stress, and higher rates of risk factors such as hypertension, cigarette smoking and obesity [1, 2]. Lower SES individuals may lack the financial capacity for preventive healthcare or treatment, increasing their CVD risk [3]. Their restricted access to healthy foods and safe physical activity environments can promote unhealthy behaviors, contributing to obesity and hypertension [4]. Chronic stress due to social inequality can also precipitate CVD through elevated inflammatory markers and sympathetic nervous system activation [5].

Arterial stiffness information holds significance as it can predict a patient’s cardiovascular risk [6, 7]. Pulse wave velocity (PWV) is the most widely employed method for measuring arterial stiffness [8]. Recently, a method for predicting PWV using age and blood pressure information, devoid of a measurement tool, was developed and validated [9]. Numerous studies have confirmed that estimated PWV (ePWV) is a robust predictor of patients’ cardiovascular risk [10–13]. Since ePWV can be acquired through a straightforward calculation formula, requiring no specific measuring equipment, it possesses high future clinical utility.

Identifying the factors that escalate arterial stiffness is crucial for risk prediction and treatment. From this perspective, the potential impact of SES on arterial stiffness might be intriguing. However, there is scant knowledge about whether SES truly influences arterial stiffness [14]. Thus, in this study, we aim to examine the association between ePWV and SES, drawing on data from a large cohort within a national study.

## Methods

### Study population

This study analyzed data from the Korean National Health and Nutrition Survey (KNHANS) conducted between 2019 and 2021. The KNHANES, conducted by The Korea Disease Control and Prevention Agency, is a nationwide survey designed to evaluate the health and nutritional status of the Korean population [15]. It gathers data on health-related behaviors, health conditions, and the nutritional intake of Koreans through a combination of interviews, physical examinations, nutritional assessments, and laboratory tests. This survey serves as a foundation for public health policy-making, monitoring risk factors for major diseases, and assessing trends in health and nutrition within the Korean population [16]. KNHANES data is publicly accessible online, allowing researchers to use it for analysis. This study initially targeted 15,407 adults aged between 20 and 80 years. Of these, 918 individuals lacking blood pressure (BP) data, 62 individuals without income data, and 888 individuals missing education level data were sequentially excluded, resulting in a total of 13,539 participants being analyzed. The flow diagram for study enrollment is shown in Supplementary Figure S1. The study protocol was reviewed and approved by the Institutional Review Board of Boramae Medical Center, Seoul, Korea (approval number: 07-2023-41). Additionally, a waiver of consent was obtained.

### Clinical data collection

Height and weight were measured simultaneously using automated equipment. Body mass index (BMI) was calculated as weight in kilograms (kg) divided by the square of height in meters (m²). Waist circumference was measured by a trained individual using a tape measure around the navel, ensuring not to compress the skin, during exhalation. Systolic and diastolic BPs were measured using an automatic BP monitor following a standardized procedure [17]. Information regarding cardiovascular risk factors including hypertension, diabetes mellitus, dyslipidemia, smoking history, and history of angina, myocardial infarction, and stroke was collected through a standardized questionnaire. Following an overnight fast, approximately 10 mL of blood was drawn from the antecubital vein to measure the levels of various laboratory parameters including hemoglobin, glucose, glycated hemoglobin, uric acid, creatinine, total cholesterol, low-density lipoprotein cholesterol, high-density lipoprotein cholesterol, and triglycerides. The glomerular filtration rate was estimated using the Modification of Diet in Renal Disease equation. Additionally, information on the use of anti-hypertensives, anti-diabetics, and anti-dyslipidemics was collected from the participants.

### ePWV calculation

The calculation of ePWV was performed according to existing literature data [9, 12, 13]. Initially, the mean BP (MBP) was calculated using the formula: MBP = diastolic BP + [0.4 × (systolic BP-diastolic BP)]. Subsequently, ePWV was computed by incorporating the patient’s age and MBP into the following equation: ePWV (m/s) = 9.587 - (0.402 × age) + (4.560 × 0.001 × age^2^) - (2.621 × 0.00001 × age^2^ × MBP) + (3.176 × 0.001 × age × MBP) - (1.832 × 0.01 × MBP) [9]. The distribution of ePWV is shown in Supplementary Figure S2.

### SES

Although several variables correspond to SES, this study focused on individual and household income, and education level as two critical indicators of SES. These factors are essential for evaluating SES, and the KNHANES provides this information. Data on monthly individual and household income, as well as education level, were obtained through a questionnaire. Individual and household income were stratified into four quartiles. Education levels were categorized into four groups: no schooling or elementary school only, middle school graduation, high school graduation, and college graduation or higher.

### Statistical analysis

Data were presented as mean ± standard deviation for continuous variables, and n (%) for categorical variables. Study subjects were divided into two groups based on the median ePWV (= 8.47 m/s), and clinical characteristics were compared. Continuous variables were compared using the Student’s t-test, while non-continuous variables were compared using the chi-square test. The ePWV values across different levels of individual and household income and education were analyzed using analysis of variance. To explore the independent associations of ePWV with household income and education level, multivariable linear regression and binary logistic regression analyses were performed. For linear regression, adjustments were made for age, sex, BMI, systolic BP, glycated hemoglobin, low-density lipoprotein cholesterol, glomerular filtration rate, and uric acid level. Binary logistic regression analysis included adjustments for age, sex, BMI, hypertension, diabetes mellitus, dyslipidemia, cigarette smoking, glomerular filtration, and uric acid level. These adjustments provided a comprehensive evaluation of the independent relationships between household income, education level, and ePWV, while accounting for a broad range of potential confounding factors. This approach is essential to gain a more accurate and detailed understanding of the association between socioeconomic factors and arterial stiffness. In the multivariable linear regression analysis, multicollinearity was assessed using the variance inflation factor (VIF), with all independent variables showing VIF values below 3 [18]. For the multivariate binary logistic regression, the dependent variable was classified according to the median value of ePWV (= 8.47 m/s). The goodness of fit for the logistic regression model was evaluated using the Hosmer-Lemeshow test, with all resulting *P* values being > 0.05 [19]. Analyses were two-tailed, and a *P* value < 0.05 was considered statistically significant. All statistical analyses were performed using SPSS version 26.0 (IBM Corp., Armonk, NY, USA).

## Results

### Clinical characteristics of study subjects

Table 1 illustrates the clinical characteristics of the patients included in the study. The mean age of the participants was 52.9 ± 16.7 years, with women constituting 57.1% of the study population. The average BMI was 24.0 ± 3.6 kg/m^2^. Both systolic and diastolic BPs fell within the normal range. Hypertension, diabetes, and dyslipidemia were present in 26.6%, 11.1%, and 22.7% of the patients, respectively. The study identified 15.7% of the participants as smokers. Additionally, 3.5% of the patients had a history of angina pectoris or myocardial infarction, and 2.6% had experienced a stroke. The majority of blood test results were within the normal range. Among the patients, 24.5% were taking antihypertensive medications, 10.5% were on diabetes medications, and 17.3% were receiving medications for dyslipidemia. Compared to individuals with low ePWV (< 8.47 m/s), those with high ePWV (≥ 8.47 m/s) exhibited unfavorable results in almost all cardiovascular clinical indicators.

**Table 1:** Clinical characteristics of study subjects

Characteristic	Total (*n* = 13,539)	Subjects with lower ePWV (< 8.47 m/s) (*n* = 6,775)	Subjects with higher ePWV (≥ 8.47 m/s) (*n* = 6,764)	*P*
Age, years	52.9 ± 16.7	39.4 ± 10.7	66.4 ± 9.1	< 0.001
Female sex	7,724 (57.1)	4,002 (59.1)	3,722 (55.0)	< 0.001
Height, cm	163 ± 9	166 ± 8	160 ± 9	< 0.001
Weight, kg	64.4 ± 12.8	65.8 ± 13.9	63.1 ± 11.4	< 0.001
Waist circumference, cm	84.2 ± 10.5	81.4 ± 10.8	87.0 ± 9.4	< 0.001
Body mass index, kg/m^2^	24.0 ± 3.6	23.7 ± 3.8	24.4 ± 3.3	< 0.001
Systolic blood pressure, mmHg	119 ± 16	110 ± 11	129 ± 15	< 0.001
Diastolic blood pressure, mmHg	74.9 ± 9.7	72.9 ± 8.5	76.9 ± 10.5	< 0.001
*Cardiovascular risk factors*				
Hypertension	3,608 (26.6)	408 (6.0)	3,200 (47.3)	< 0.001
Diabetes mellitus	1,509 (11.1)	220 (3.2)	1,289 (19.1)	< 0.001
Dyslipidemia	3,069 (22.7)	602 (8.9)	2,467 (36.5)	< 0.001
Current smoking	2,119 (15.7)	1,229 (1801)	890 (13.2)	< 0.001
Previous angina or myocardial infarction	472 (3.5)	31 (0.5)	441 (6.5)	< 0.001
Previous stroke	354 (2.6)	32 (0.5)	322 (4.8)	< 0.001
*Laboratory findings*				
Hemoglobin, g/dL	13.8 ± 1.5	13.9 ± 1.6	13.7 ± 1.4	< 0.001
Fasting glucose, mg/dL	101 ± 22	96.3 ± 18.2	107 ± 25	< 0.001
Glycated hemoglobin, %	5.83 ± 0.82	5.58 ± 0.66	6.08 ± 0.89	< 0.001
Uric acid, mg/dL	5.13 ± 1.38	5.16 ± 1.40	5.10 ± 1.36	0.016
Glomerular filtration rate, mL/min/1.73m^2^	91.1 ± 19.3	97.5 ± 17.5	84.6 ± 18.9	< 0.001
Total cholesterol, mg/dL	190 ± 38	194 ± 35	186 ± 41	< 0.001
Low-density lipoprotein cholesterol, mg/dL	115 ± 36	122 ± 36	108 ± 36	< 0.001
High-density lipoprotein cholesterol, mg/dL	52.2 ± 12.8	54.2 ± 13.0	50.2 ± 12.2	< 0.001
Triglyceride, mg/dL	128 ± 97	120 ± 94	136 ± 98	< 0.001
*Medications*				
Anti-hypertensives	3,221 (24.5)	355 (5.2)	2,966 (43.8)	< 0.001
Anti-diabetics	1,426 (10.5)	203 (3.0)	1,233 (18.1)	< 0.001
Anti-dyslipidemics	2,340 (17.3)	378 (5.6)	1,962 (29.0)	< 0.001

Numbers are expressed as mean ± standard deviation or n (%). ePWV, estimated pulse wave velocity

### Associations of household incomes and education levels with ePWV

**Fig. 1:**
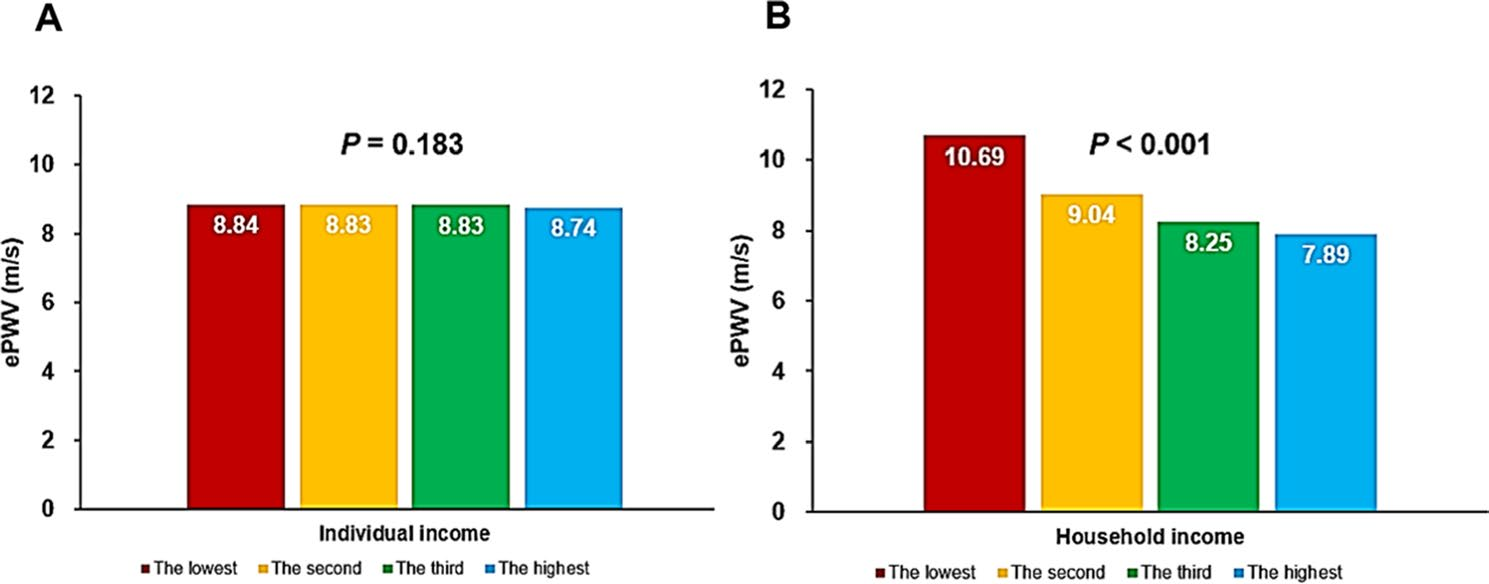
Associations of individual (**A**) and household (**B**) income with ePWV. ePWV, estimated pulse wave velocity

**Fig. 2:**
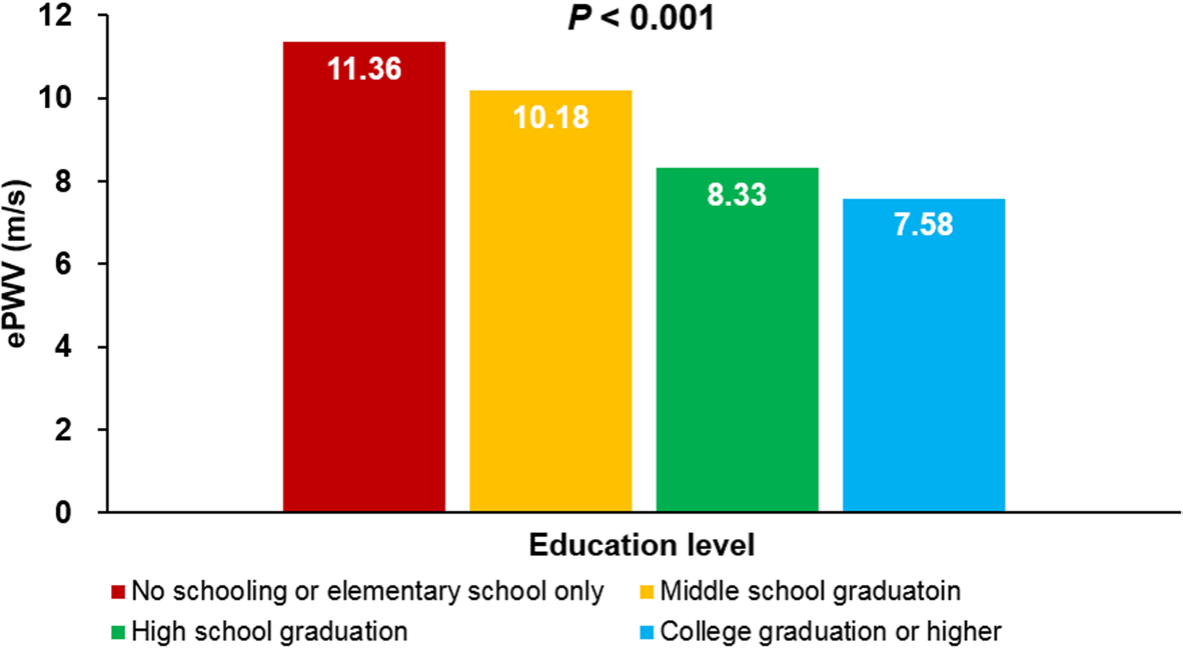
Association between education level and ePWV ePWV, estimated pulse wave velocity

The ePWV did not vary significantly across groups categorized by individual income levels (*P* = 0.183). However, there was a noticeable trend of gradual ePWV reduction with increasing household income levels (*P* < 0.001) (Fig. 1). Additionally, the ePWV value demonstrated a significant negative correlation with higher education levels, indicating that ePWV decreased in groups with greater educational attainment (*P* < 0.001) (Fig. 2). In the multiple linear regression analyses, both household income (*β* = -0.055; *P* < 0.001) and education level (*β* = -0.076; *P* < 0.001) were found to be negatively associated with ePWV, even after adjusting for potential confounders (Table 2). Moreover, multiple binary logistic regression analyses revealed that lower household income (comparing the lowest to the highest quartile: odds ratio [OR] = 3.13; 95% confidence interval [CI] = 2.58–3.79; *P* < 0.001) and lower education level (no schooling or only elementary school vs. college graduation or higher: OR = 11.42; 95% CI = 8.68–15.02; *P* < 0.001) were associated with a higher likelihood of having an ePWV ≥ 8.47 m/s, even after accounting for the confounding effects of major clinical variables (Table 3).

**Table 2:** Linear regression analyses showing the associations of ePWV with household income and education level

	Unadjusted				Adjusted*			
	*β*	Standard error	t	*P*	*β*	Standard error	t	*P*
Household income	-0.428	0.016	-55.16	< 0.001	-0.055	0.011	-8.773	< 0.001
Education level	-0.633	0.013	-95.20	< 0.001	-0.076	0.013	-10.197	< 0.001

*Following clinical covariates were controlled during the analysis: age, sex, body mass index, systolic blood pressure, glycated hemoglobin, low-density lipoprotein cholesterol, glomerular filtration rate and uric acid. ePWV, estimated pulse wave velocity

**Table 3:** Binary logistic linear regression analyses showing the associations of higher ePWV (≥ 8.47 m/s) with household income and education level

	Unadjusted		Adjusted*	
	OR (95% CI)	*P*	aOR (95% CI)	*P*
*Household income*				
The highest	1	-	1	-
The second	1.30 (1.18–1.43)	< 0.001	1.32 (1.13–1.55)	< 0.001
The third	2.44 (2.22–2.69)	< 0.001	1.99 (1.69–2.34)	< 0.001
The lowest	8.30 (7.37–9.33)	< 0.001	3.13 (2.58–3.79)	< 0.001
*Education level*				
College graduation or higher	1	-	1	-
High school graduation	2.18 (2.00-2.38)	< 0.001	1.34 (1.17–1.54)	< 0.001
Middle school graduation	15.18 (12.97–17.75)	< 0.001	3.17 (2.55–3.95)	< 0.001
No schooling or elementary school only	90.02 (71.87-112.75)	< 0.001	11.42 (8.68–15.02)	< 0.001

*Following clinical covariates were controlled during the analysis: age, sex, body mass index, hypertension, diabetes mellitus, dyslipidemia, cigarette smoking, glomerular filtration rate and uric acid. ePWV, estimated pulse wave velocity; OR, odds ratio; CI, confidence interval; aOR, adjusted odds ratio

## Discussion

### Study summary

We aimed to investigate the link between arterial stiffness and SES in a substantial cohort of Korean adults, utilizing data from the KNHEANS. The principal finding of our research indicated that while arterial stiffness, as measured by ePWV, was not associated with individual income levels, it showed a significant correlation with household income and the individual’s level of education. Specifically, lower household income and lower education levels were associated with higher ePWV values. This relationship remained statistically significant in both univariate analyses and multivariate analyses that adjusted for the influence of major clinical variables. Although there have been some studies suggesting a link between arterial stiffness and SES, to our knowledge, none have specifically explored the relationship between ePWV and both household income and education level.

### Previous studies on the association between SES and arterial stiffness

In a longitudinal study of 3,836 British individuals, the gradual increase in aortic PWV measured at five-year intervals was more pronounced among those with lower employment grades, low household income, and low educational attainment [20]. Poulakka et al. assessed arterial PWV and carotid distensibility in 2,566 participants, and found that higher SES in childhood was linked to lower arterial stiffness in adulthood [21]. Additional research on 1,761 individuals from the same cohort revealed that high levels of neighborhood deprivation during both childhood and adulthood were associated with increased PWV in later life, even after adjusting for age, sex, and place of birth [22]. Another study, involving 3,342 participants aged 67–89 years without CVD from the Atherosclerosis Risk in Communities (ARIC) study, demonstrated that higher education and income levels were correlated with lower carotid-femoral PWV (cfPWV) values [23]. Moreover, in the ARIC study, analysis of 10,091 men and women aged 45–64 years revealed that lower educational levels were linked to reduced carotid artery pulsatility [24]. A few years prior, we analyzed data from 13,004 Korean adults from the 2018 KNHANES and found a significant negative correlation between household income level and pulse pressure [25]. Furthermore, our analysis of 8,929 individuals undergoing CVD evaluation revealed that baPWV values were significantly higher among Medical Aid recipients compared to National Health Insurance beneficiaries [14]. Our current study also identified that elevated ePWV was associated with lower household income and lower educational levels, findings that align with those of previous research. These results offer further evidence supporting the influence of SES on arterial stiffness.

### Underlying mechanisms

The precise mechanisms underlying the association between SES and arterial stiffness remain unclear, but the relationship is complex and influenced by a myriad of direct and indirect factors. Several hypotheses have been proposed to explain this association. Firstly, lower SES may hinder timely access to healthcare services, leading to a higher prevalence and incidence of cardiovascular risk factors such as hypertension, diabetes, and dyslipidemia, all of which can contribute to increased arterial stiffness [1]. Furthermore, individuals with lower SES often exhibit unhealthy lifestyle habits, including smoking, excessive alcohol consumption, high intake of fast food, and reduced physical activity, leading to a higher prevalence of obesity—factors known to escalate arterial stiffness [26]. Chronic stress, more common among those facing financial hardships, job insecurity, and poor living conditions associated with lower SES, can trigger the release of stress hormones like cortisol, resulting in vascular inflammation and heightened arterial stiffness [27]. Environmental factors also play a role; lower SES is frequently associated with living in areas of higher pollution and exposure to toxic substances, along with limited access to green spaces. These conditions can lead to oxidative stress and inflammation, further exacerbating arterial stiffness [28]. Additionally, education, a component of SES, influences health literacy, impacting an individual’s capacity to manage health risks, comply with medical advice, and make informed health decisions. Higher levels of education are linked to better health outcomes and potentially reduced arterial stiffness.

### Individual vs. household income

In our study, ePWV was not correlated with personal income but showed a significant correlation with household income. While there has been limited research specifically addressing this observation, we propose several possible explanations. Household income represents the collective income of all members within a household, thus providing a more comprehensive reflection of the household’s overall economic situation. This collective measure may have a more substantial impact on health than individual income, influencing the health status of all household members through various factors such as dietary habits, living conditions, and healthcare access. A higher household income enables better living standards, including higher-quality diets, regular health check-ups, and improved healthcare access. These elements are crucial in preventing and managing conditions that influence arterial stiffness, like hypertension, diabetes, and hyperlipidemia. Additionally, families with higher household incomes often have higher education levels, which can lead to better health literacy, access to health information, and healthier lifestyle choices. Educated families are typically more informed about health issues and more proactive in adopting preventive health measures. Conversely, lower household income environments are often associated with increased psychological stress, which can directly impact arterial stiffness through stress-induced hormonal changes. This stress can lead to arterial stiffening and elevated blood pressure [27]. Furthermore, lower-income households are more likely to face adverse environmental conditions, such as exposure to air and water pollution and substandard living environments, all of which can detrimentally affect health and contribute to increased arterial stiffness [28]. These considerations suggest that household income has a more pronounced effect on health, particularly arterial stiffness, than individual income. Nonetheless, further research is required to more precisely elucidate this relationship.

### The value of ePWV

The significant advantage of ePWV lies in its ability to be calculated using only age and BP [9]. These two parameters are among the most fundamental and easily measurable health indicators available. Consequently, ePWV can be conveniently utilized across various medical settings, from hospitals to clinics. The absence of a requirement for special equipment means that ePWV offers a cost-effective method for assessing the risk of CVD. This feature is especially beneficial in regions with limited medical resources or in lower-income countries. The capacity to estimate ePWV with just age and BP proves exceptionally valuable for conducting large-scale population surveys or research. It facilitates the identification of demographic trends associated with cardiovascular health and generates vital data for the formulation of health policies and intervention strategies. Therefore, the calculation and application of ePWV enhance the accessibility of CVD risk assessment and management on a wider scale, positioning it as a crucial instrument for advancing cardiovascular health worldwide.

### Clinical implications

Our study provides further evidence on the impact of SES on arterial stiffness, suggesting that low SES may adversely affect cardiovascular health. It underscores the necessity of early cardiovascular risk assessments and interventions, especially for individuals with lower household incomes or education levels. The findings advocate for the customization of health promotion and prevention programs to consider socioeconomic backgrounds, indicating the need for specialized health improvement initiatives targeting those with lower SES. Moreover, the study emphasizes the crucial role of education in promoting cardiovascular health. It calls for health-related education programs and campaigns to increase awareness about the benefits of a healthy lifestyle and to motivate adoption across all societal strata. Addressing the effects of low household income and education level on cardiovascular health requires a multidisciplinary strategy, involving collaboration among professionals from the medical, educational, and social welfare sectors. This comprehensive approach is vital for developing public health policies that not only consider socioeconomic factors but also aim to mitigate health disparities among the economically and educationally disadvantaged.

### Study limitations

Our study has several limitations. Firstly, we performed a cross-sectional analysis, and thus, our results do not imply a causal relationship between SES and arterial stiffness. Secondly, the BP measurement method used in KNHANES from 2019 to 2021 varied slightly each year. This variation might introduce some inconsistency in the ePWV values for each year. However, even when separately analyzing subjects from 2019 to 2020 (*n* = 9,215), whose BP was measured using relatively similar methods, the independent association between SES and ePWV was also observed (Supplementary Table S1). Thirdly, in the univariable analysis, almost all clinical indicators differed between individuals with low and high ePWV. However, not all of these indicators could be adjusted for in the multivariable analysis. We performed multivariable adjustments mainly on indicators with relatively well-known associations with arterial stiffness. Therefore, although there may be variables we are not familiar with, we must keep in mind the possibility that their confounding effects may have influenced the results. Lastly, our study population was restricted to the Korean general population, which may limit the generalizability of our results to other ethnic groups.

## Conclusions

In Korean adults, lower household income and education levels were found to be associated with increased ePWV, offering further evidence of the impact of SES on arterial stiffness. This underscores the heightened cardiovascular risk among individuals with lower SES, highlighting the need for targeted intervention.

## Electronic supplementary material

Below is the link to the electronic supplementary material.

Supplementary Material 1

## Data Availability

The KNHANES data is publicly accessible on the Korea Disease Control and Prevention Agency website (https://www.kdca.go.kr/).

## Abbreviations

ARIC: Atherosclerosis Risk in Communities
BMI: Body mass index
BP: Blood pressure
CfPWV: Carotid-femoral pulse wave velocity
CVD: Cardiovascular disease
Epwv: Estimated pulse wave velocity
KNHANS: Korean National Health and Nutrition Survey
MBP: Mean blood pressure
PWV: Pulse wave velocity
SES: Socioeconomic status
VIF: Variance inflation factor
