# Supplementary material for: The association between arterial stiffness and socioeconomic status: a cross-sectional study using estimated pulse wave velocity
Source: Clin Hypertens. 2024 Oct 1;30:26. doi: 10.1186/s40885-024-00284-7 (PMC11443864; doi:10.1186/s40885-024-00284-7)
Supplement: Supplementary file 1 — Supplementary Material 1 [file 40885_2024_284_MOESM1_ESM.docx]

**Supplementary Data**

**Supplementary Figure S1. Flow diagram for study enrollment**


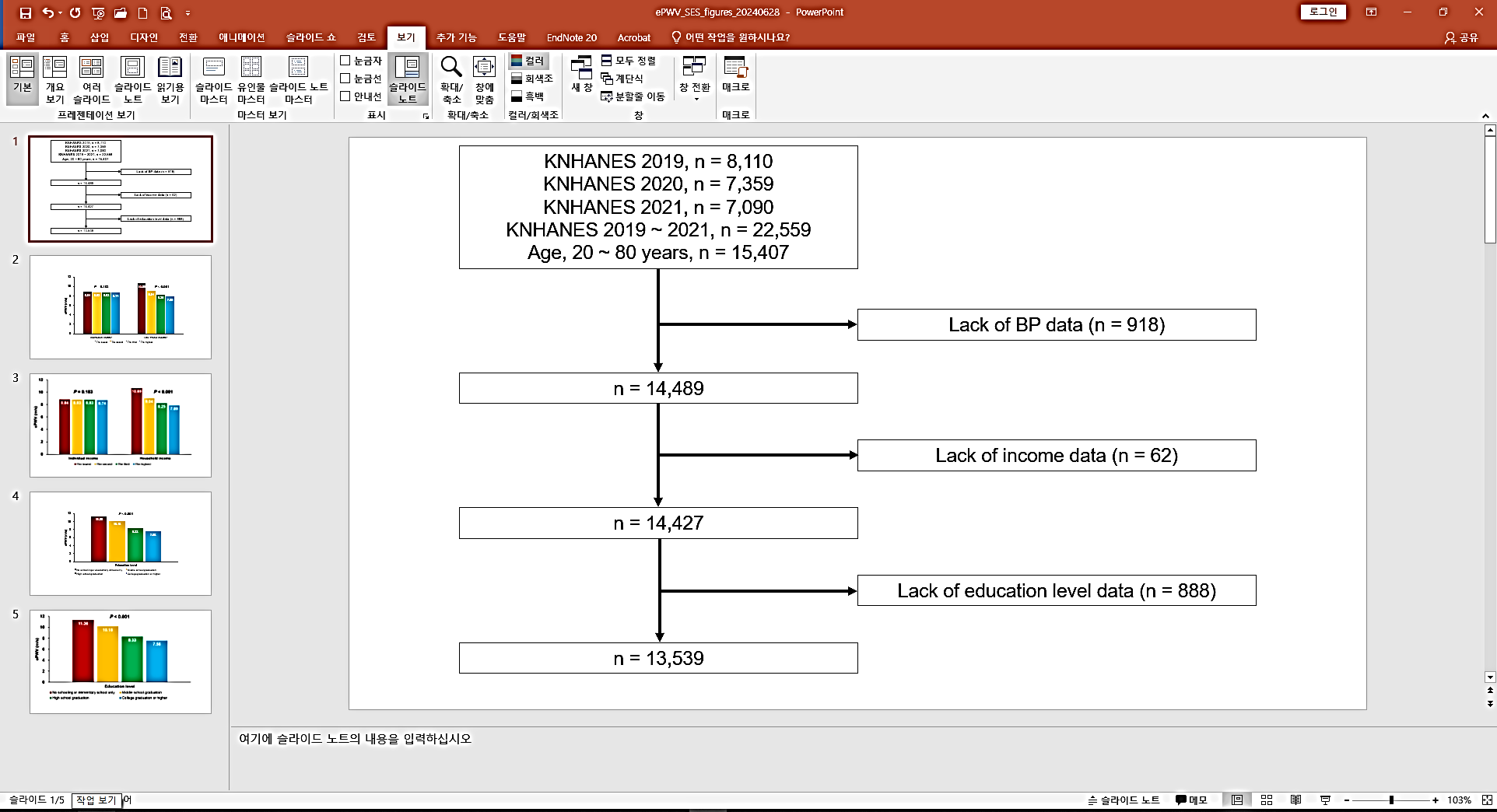


KNHANES, Korean National Health and Nutritional Examination Survey; BP, blood pressure.

**Supplementary Figure S2. ePWV distribution of study subjects**

**
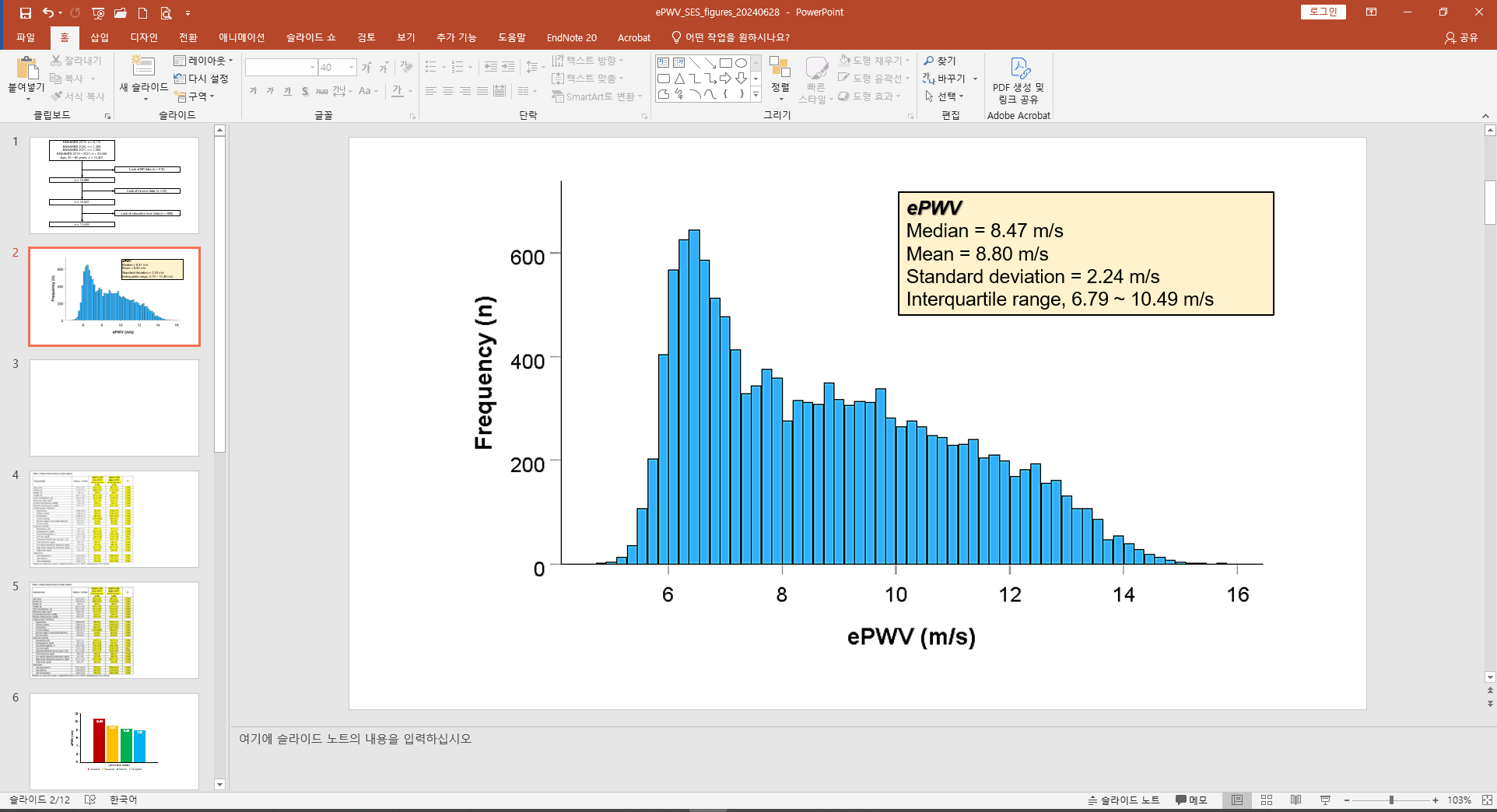
**

ePWV, estimated pulse wave velocity.

**Supplementary Table S1. Multiple linear regression analyses showing the associations of ePWV with household income and education level among participant between 2019 and 2020 (n = 9,215)**

|  | ***β*** | **t** | ***P*** |
| --- | --- | --- | --- |
| Household income | -0.055 | -8.880 | < 0.001 |
| Education level | -0.056 | -9.044 | < 0.001 |

Following clinical covariates were controlled during the analysis: age, sex, body mass index, systolic blood pressure, glycated hemoglobin, low-density lipoprotein cholesterol, glomerular filtration rate and uric acid. ePWV, estimated pulse wave velocity.
